# Between words and practice: a qualitative exploration of physical literacy understanding and application

**DOI:** 10.3389/fspor.2025.1703265

**Published:** 2025-10-16

**Authors:** Ivan Curovic, David Grecic, Aleksandar Lekic, Dejan Suzovic, Lazar Toskic, Igor Curovic

**Affiliations:** ^1^ Institute of Coaching & Performance (ICaP), University of Lancashire, Preston, United Kingdom; ^2^ Institute for Behaviour, Sport and Rehabilitation, University of Lancashire, Preston, United Kingdom; ^3^ School of Health, Social Work and Sport, University of Lancashire, Preston, United Kingdom; ^4^ Faculty of Sport and Physical Education, University of Belgrade, Belgrade, Serbia; ^5^ Faculty of Sport and Physical Education, University of Pristina in Kosovska Mitrovica, Leposavic, Serbia; ^6^ Faculty of Sport, University “Union -Nikola Tesla”, Belgrade, Serbia; ^7^ Judo Club Partizan, Belgrade, Serbia

**Keywords:** pedagogy, teaching, sport, physical activity, lifelong participation, children, physical education

## Abstract

Physical literacy (PL) has become an internationally recognised framework for promoting lifelong physical activity, yet its interpretation and application remain inconsistent across different contexts. In Serbia, where Physical Education (PE) is historically performance-driven and PL is absent from curricula and policy, little is known about how teachers understand and apply the concept. This qualitative study explored the philosophies and practices of Serbian PE teachers (in-service, *n* = 20; pre-service, *n* = 11) through semi-structured interviews applying deductive thematic analysis in relation to the core elements of PL informed by the IPLA's definition (2017). Findings show that although PL was largely unheard of, it was commonly described in ways that captured its holistic dimensions of health, motivation, and lifelong engagement, albeit with a predominant focus on physical competence. Participants expressed positive aspirations for PE as a subject, frequently emphasising values such as pupil wellbeing, enjoyment, and long-term habit formation. However, practical accounts were dominated by traditional, teacher-led lessons focused on drills, discipline, and technical performance, with student autonomy and creativity rarely depicted. For pre-service teachers, discipline emerged as a central expectation, with compliance and order described as prerequisites for learning with few participants articulating student-centred practices. Overall, the findings highlight a clear gap between PL-aligned holistic philosophy and traditional practice. Addressing this gap through curriculum reform, teacher education, and professional development is crucial if PL is to be meaningfully embedded in Serbian PE and positively influence pupils’ confidence, autonomy, and lifelong activity.

## 1 Introduction

Physical Literacy (PL) has emerged over the past two decades as a key organising concept for promoting lifelong engagement in physical activity, health, and well-being (1, 2). Despite its growing popularity, PL remains a complex and contested concept (3, 4), encompassing multiple factors including physical competence, motivation, confidence and knowledge (5), all integrated and developed through the individual's lived movement experiences across life (6–8). Moreover, Young et al. (9) proposed PL as a holistic “multiverse” that encapsulates health promotion, motor competence and embodied identity. Despite slight fluctuations in definitions and diverse interpretations across countries regarding its scope and practical application (3, 4), major international organisations such as the World Health Organization (WHO) and UNESCO advocate for PL as a foundational component of physical activity and Quality Physical Education (QPE) (10), recognising its potential to frame active lifestyles from childhood through adulthood (11). Global interest in PL is reflected in its integration into national curricula and professional standards in multiple developed countries across the world (12). Preliminary findings from the EUROPLIT study (10), however, highlighted significant disparities in the integration of PL across European nations, particularly in countries with limited prior research activity in this area. Indeed, only 3 countries of the 40 recently surveyed had PL as the “dominant concept” in their PE curriculum with many still having no verbatim mention of it within their documentation (13).

Notably, Serbia is absent from many pan-European studies and currently has no official PL-aligned association to promote or guide its integration into education and sport systems. PE in Serbia is shaped by a complex mix of educational traditions and cultural expectations, with a persistent focus on performance-driven methods influenced by the nation's long-standing sporting successes (14, 15). Consequently, there is currently no nationally endorsed definition of PL. For the purposes of this study, therefore, we adopt the International Physical Literacy Association's (IPLA) definition that describes PL as “the motivation, confidence, physical competence, knowledge and understanding to value and take responsibility for engagement in physical activities for life” (5). That said, our interpretive framework is also informed by the holistic dimensions of other prominent models, such as the Australian framework's emphasis on social and cognitive domains (8). This is especially important due to the Serbia's national PE curriculum's focus on physical performance, technical skills, and memorisation of sport-related knowledge (16) which could restrict opportunities for adopting more holistic, pupil-focused approaches that reflect PL's broader dimensions. These curricular challenges are compounded by the marginal position of PE in schools, where the profession works within outdated facilities, limited resources, and scarce professional development opportunities (15) despite national policy documents acknowledging the value and importance of physical activity (17).

The PL concept is philosophically grounded in monism, phenomenology, and existentialism (1), thereby rejecting the Cartesian mind-body dualism, viewing the individual as an integrated whole. Phenomenology in this context emphasises the importance of subjective, lived experiences of *movement*, while existentialism highlights the role of individual choice and responsibility in finding meaning through physical activity (1, 4). They place PL as a unique, holistic construct that goes beyond mere physical competence, focusing on the personal meaning and value of movement across the lifespan (5, 7, 8). This philosophical stance stands in contrast to the performance-oriented traditions shaping Serbian PE. Without formal curriculum integration or coordinated advocacy, the extent to which PL is understood, valued and applied in schools depends largely on the knowledge, beliefs, and practices of those teaching PE as a core subject (18). Little is known, however, about Serbian PE teachers' awareness and interpretation of PL, or whether their current approaches align with its core principles. Such a knowledge gap limits the capacity to design evidence-informed curriculum reforms and professional development strategies that can leverage PL to enhance QPE and promote lifelong engagement in physical activity. The present study addresses this gap by exploring Serbian in-service and pre-service PE teachers' PL knowledge and understanding, examining whether their practices align with PL principles and elements, and identifying developmental needs for future professional development. Reflecting the wider international dialogue on PL and the specific circumstances of Serbia's education system, the study explores current position of PL within the nation's PE and considers possible directions for its future promotion. The study's ultimate aim is to uncover new knowledge about the teaching profession that can inform how PE in Serbia can facilitate children's positive dispositions to movement and opportunities in schools that are meaningful, inclusive, and sustainable for all pupils while accounting for the country's cultural-contextual considerations (19).

## 2 Materials and methods

### 2.1 Study design

This study aimed to explore the knowledge, beliefs, and practices of Serbian pre-service and in-service PE teachers in relation to PL, examining the extent to which their professional philosophies and pedagogical approaches aligned with its core elements. We employed qualitative design, using semi-structured interviews as the primary method of data collection with an initial deductive approach. Specifically, the analytical framework was informed by the four elements of the IPLA's definition of PL—motivation, confidence, physical competence, and knowledge (5), which acted as *a priori* categories to guide the coding and thematic structuring of data. Philosophically, the study was underpinned by an interpretivist paradigm, recognising that teachers' accounts reflect socially constructed understandings shaped by their personal experiences, cultural context, and professional environment (18). This was further situated within a relativist ontology and constructivist epistemology (20), acknowledging that there is no single “truth” about PL or PE practice, but rather multiple situated interpretations shaped by context and experience. Ethical approval for the study was obtained from the Ethics panel of the University of Lancashire and all procedures complied with institutional and professional ethical guidelines.

### 2.2 Participants

Twenty PE teachers (12 elementary, 8 secondary) and 11 pre-service teachers participated in the study. This sample was purposefully selected in order to provide a broad range of PE perspectives based on a wide range of background and experiences. Serbia itself does not have a formal teaching qualification requirement beyond graduation from the Sport and PE university degrees offered from its universities. The sample therefore represents the range of PE teachers who can contribute to the profession's knowledge base and professional learning community. The participants were recruited from four Serbian cities through the authors' professional network. Eligibility criteria required in-service teachers to hold a PE degree qualification and have a minimum of five years teaching experience at either the elementary or secondary level. This criterion ensured that participants had both formal professional preparation and sufficient teaching practice to articulate views on their educational philosophy and pedagogical approaches. Pre-service teachers were eligible if they were in their final year of university studies (thus had completed their practical teaching placements) or had graduated within one year prior to the interviews. Participant demographics, including age, sex, level of teaching, curriculum experience, and years of practice, are presented in Tables 1, 2, respectively.

**Table 1:** Physical education in-service teachers’ demographic information (*n* = 20, mean age 42 years, mean experience 12 years).

Teacher	Sex	Age	School workplace	Curriculum experience	Years of experience
T1	M	43	Secondary	National & international	11
T2	M	52	Secondary	National	17
T3	F	37	Secondary	National	7
T4	M	51	Secondary	National	21
T5	M	39	Elementary	National & international	12
T6	F	40	Elementary	National & international	5
T7	M	45	Secondary	National	13
T8	M	52	Elementary	National	15
T9	M	51	Elementary	National	22
T10	M	36	Elementary	National	8
T11	M	44	Elementary	National & international	13
T12	M	48	Elementary	National	20
T13	M	36	Elementary	National	8
T14	F	35	Elementary	National	6
T15	F	42	Elementary	National	12
T16	M	35	Elementary	National	5
T17	F	55	Secondary	National	31
T18	M	42	Secondary	National	15
T19	M	40	Elementary	National	8
T20	M	36	Secondary	National & international	5

**Table 2:** Physical education pre-service teachers’ demographic information (*n* = 11, mean age 23 years).

Teacher	Sex	Age	School workplace	Curriculum experience	Years of experience
T21	F	23	University placement only	Placement	0
T22	M	23	Sport school	Elementary	2
T23	M	22	Sport school	Elementary	3
T24	F	22	University placement only	Placement	0
T25	M	22	Ski school	Elementary	1
T26	M	21	University placement only	Placement	0
T27	M	23	Part-time PE teacher	Elementary	1
T28	F	23	University placement only	Placement	0
T29	F	23	Gymnastic school	Elementary	2
T30	F	24	University placement only	Placement	0
T31	M	23	University placement only	Placement	0

### 2.3 Data collection

Data were collected through individual, semi-structured interviews. Each interview was designed to examine participants' philosophies, values, and practices regarding PE and PL core ideas. Guiding questions included prompts such as “How would you describe your overall teaching philosophy?”, “Have you heard of the concept of Physical Literacy?”, “What would a typical teaching session look like for your pupils?”, “How would you like your relationship with your (future) students/clients/athletes to develop?”, “What do you believe should be the main focus of development in physical education or sport classes for children?”. Additional follow-up questions probed teachers' descriptions of classroom practice and decision-making, encouraging them to provide concrete examples of lesson structures, teaching strategies, and intended learning outcomes. All interviews were conducted face-to-face in the Serbian language, recorded with participant consent, and later transcribed verbatim. Transcripts were subsequently translated into English for analysis and reporting. Each participant was anonymised and assigned a code (T1–T31). Interviews lasted between 35 and 55 min (mean duration 45 min).

### 2.4 Data analysis

The analytical process was initially deductive, guided by the four core elements of PL (motivation, confidence, physical competence, and knowledge) (5), which provided the coding framework. First, transcripts were read and re-read by the authors to achieve immersion in the dataset. Segments of text were then coded under the relevant PL elements using NVivo (version 14). Within each element, patterns with recurring ideas, experiences, or interpretations were identified, leading to the generation of subthemes. The research team engaged in iterative discussions, acting as “critical friends” (21) to challenge assumptions, refine coding decisions, and consider alternative interpretations. This collaborative process ensured that the analysis did not merely categorise responses mechanically, but rather sought to capture the nuanced ways in which teachers' philosophies and practices resonated with or diverged from the principles of PL. In particular, discrepancies between stated philosophies and described classroom practices were carefully noted and incorporated into the later thematic narrative.

Following deductive codes being derived from the established theoretical framework, the analysis applied an interpretive lens. In practice, this allowed for inductive insights to emerge within the deductive structure. During the analytical process, it became evident that participants' descriptions often integrated four PL elements (5) in a holistic manner. For instance, teachers frequently discussed motivation and confidence as intertwined psychological constructs, and physical competence was regularly presented as the foundation for both. Therefore, while the initial coding respected the distinct IPLA categories (5), the subsequent thematic analysis logically grouped these codes into broader, integrated themes that better reflected the participants' lived experiences and the holistic manifestations of PL. This approach allowed us to remain faithful to the deductive framework while capturing the nuanced and interconnected ways in which PL principles are understood and enacted by practitioners. Concretely, under the element of knowledge, codes related to health education, lifestyle choices, and lifelong application of skills were clustered together; under confidence, codes reflecting encouragement, motivation, and self-belief were grouped. This combination of a deductive structure with an inductive, interpretive process within each theme ensured that the analysis was both theoretically informed and firmly grounded in the participants' experiences.

### 2.5 Trustworthiness

Given the study's interpretivist positioning, trustworthiness was addressed through reflexivity, transparency, and methodological coherence. All members of the research team were current or former PE teachers or university professors with professional experience across both Serbian and international contexts. While this insider perspective provided cultural familiarity and contextual sensitivity, it also required reflexive awareness of potential biases. The team engaged in ongoing reflexive dialogue throughout the research process, explicitly considering how their experiences and values might shape interpretation. Transparency was achieved by maintaining a clear audit trail of coding decisions, theme development, and analytic discussions. Credibility was strengthened through investigator triangulation, with multiple authors reviewing and discussing the coding and thematic structure. Transferability was addressed by providing rich descriptions of the Serbian PE context, participant demographics, and illustrative quotes to allow readers to judge the applicability of findings to other settings. Dependability was enhanced by adhering consistently to the deductive PL framework as the organising structure for analysis.

Aligned with Tracy's (22) eight “big tent” criteria for qualitative quality, the study sought to demonstrate: a worthy topic (addressing the underexplored state of PL in Serbian PE); rich rigour (achieved through a robust dataset and theoretically informed analysis); sincerity and credibility (through reflexive engagement and critical dialogue); resonance (by situating findings in the wider international discourse on PL); significant contribution (offering new insight into teachers' understandings and practices in an under-researched national context); and meaningful coherence (ensured by clear alignment between philosophical stance, methodological design, and analytical approach).

## 3 Results

The results for in-service teachers are presented first, followed by pre-service teachers, with a comparative discussion integrated into the latter section. No substantial differences in understanding or practice were identified between teachers working in elementary vs. secondary schools, with the primary differentiating factor being exposure to international curricula.

### 3.1 In-service teachers

This section presents findings from the qualitative interviews with Serbian PE teachers, deductively—inductively analysed to produce three themes: Holistic Philosophy and PL; Enhancing Pupils’ Knowledge and Understanding for Autonomy; and Improving Pupils' Confidence and Competence. Table 3 represents the thematic categories created through this analysis with associated subthemes and illustrative quotations from the interviewed teachers.

**Table 3:** Themes, sub-themes, and illustrative quotes in relation to physical literacy elements among Serbian physical education teachers.

Theme	Sub-theme	Illustrative quotes
Holistic philosophy and physical literacy	Holistic intentions	“My overall philosophy is to bring sport closer to children, helping them not only improve their physical fitness but also correct postural issues, and importantly, enhance social competence” (T1)
		“My overarching philosophy as a PE teacher is centred on promoting healthy lifestyle habits, influencing students to better adapt to various sporting activities, enhancing their social skills and motivation towards positive character traits, ultimately aiming to develop them into good individuals” (T17)
	Philosophical alignment with physical literacy concepts	“I understand its (physical literacy) importance in developing individuals who are not only physically competent but also confident and motivated to participate in lifelong physical activity. Promoting physical literacy in PE helps students build a strong foundation for overall well-being” (T5)
		“I want students to love movement, see PE as more than exercise, and understand how it can make them healthier and happier individuals.” (T15) “I haven't heard of the concept of physical literacy, but I would characterise it as motor literacy. The ability of students to master and understand all motor tasks” (T3)
Enhancing Pupils’ Knowledge and Understanding for Autonomy	Health and lifelong application	“Short-term I focus on mastering skills, but medium and long term I want them to apply these in everyday life” (T9)
		“I explain the importance of warm-up and recovery so they can continue exercising safely throughout their lives” (T12)
		“…I gave them choice and reflection opportunities… they take real ownership of their activity” (T11)
	Knowledge beyond sport	“I use discussions about nutrition and lifestyle choices as part of PE” (T14)
Improving Pupils’ Confidence and Competence	Motivation and encouragement	“I encourage them with my own enthusiasm and energy in lessons” (T18)
		“Sometimes I focus on weaker students first, to make them feel capable before pushing further” (T2)
		“I use positive reinforcement and celebrate every step forward.” (T15)
	Competence and skill development	“I broaden my students’ competencies, (have them) adapt them to new challenges from various sports, and develop (their) physical skills” (T8)
		“I strive to develop their motor skills as effectively as possible, so that they can better manage and be more competitive in participating in future sports” (T3)

A consistent interview feature was that many teachers had not formally encountered the term “PL” during their training or professional practice. Nevertheless, almost all were able to describe some elements consistent with PL, such as confidence, competence, and motivation to be active throughout life. Yet, only a small number, predominantly those exposed to international curricula, were able to define PL with precision and link it explicitly to practice. For example, one teacher explained that the principles of PL became clear only once he went through the International Baccalaureate curriculum guides and workshops, while another described linking health, creativity, and student independence into their classes as a result of concepts learned during his international teaching days. A few national teachers also gave fuller accounts of PL, describing it as an individual's ability to move actively and effectively while understanding the importance of physical activity for health and well-being. Across the broader sample, however, there was a striking discrepancy between the theoretical explanations and practical lesson descriptions. While most teachers talked about holistic, pupil-centred approaches, such as wanting to build lifelong habits, promote social development, or enhance motivation, the majority described lessons in the traditional “phased” structure of warm-up, skill drills, and an end-game (Table 4). These were heavily teacher-led and focused on technical performance with limited scope for pupil ownership or creativity. As teacher T9 described, “The main part of the lesson is dedicated to the content of the teaching topic, which we cover through repetition and focus on the technique of execution.” Notably, the internationally experienced teachers more consistently aligned their philosophies with practice, describing student-centred methods that encouraged autonomy, inquiry, and reflection.

**Table 4:** (Mis)alignment between theoretical perspectives on physical literacy and practical lesson descriptions among Serbian physical education teachers (note: each teacher identified at least some elements of the concept).

Category	Illustrative quotes	Teachers
Aligned with the actual practice	“…I also try to incorporate elements of choice and autonomy, allowing students to make some decisions about the activities or challenges they want to pursue” (T11)	T1, T5, T11, T20
	“…I strive to stimulate them to show some independency and generate ideas, thereby practising creativity which they apply later” (T20)	
	“…I want them to understand movement as something fun and useful for their lives, so I often design activities where they can apply ideas and solve problems, not just copy technique” (T5)	
Partially aligned with the actual practice	“…I try to create classes with the relaxing effect for mind because my students are already too overwhelmed with other subjects and they need a certain vent to emotionally and cognitively recharge” (T12)	T12, T13
	“Depending on the main part, I would emphasise muscle groups to be warmed up properly before. Second part of the main phase is some game or competition, and ending phase is summary of impressions” (T13)	
Misaligned with the actual practice	“The main part of the lesson is dedicated to the content of the teaching topic, which we cover through repetition and focus on the technique of execution. I leave the final part of the lesson for games that boost the atmosphere” (T9)	T2, T3, T4, T6, T7, T8, T9, T10, T14, T15, T16, T17, T18, T19
	“An introductory phase starts with a full-body warm-up, explosive, multi-joint exercises, sprints, squats, setups, marines. The main phase focuses on the specific lesson unit, and the final phase includes games they want to play” (T4)	
	“I can't say I’m doing something different from the things we learned at university… Warmup part is running in circles or some relay games, then mobility-based exercises. After the main phase, if they did it well, they get 10 minutes of free time to play the sport related to the class theme. Cool down is for stretching and reflection” (T16)	

When discussing the promotion of knowledge and understanding for lifelong autonomy, many teachers described PE as a subject that should nurture broader personal development rather than focus solely on technical performance. Some spoke about using PE to guide pupils towards clubs or recreational activities appropriate for them, while others described integrating discussions about nutrition and lifestyle into their teaching. For instance, T14 stated, “I use discussions about nutrition and lifestyle choices as part of PE”. This integrated health education into lessons was often highlighted by the teachers, though without clear articulation on its application. Several emphasised their role in building not just physical skills, but also healthy and active individuals who understand the importance of physical activity. Even those unfamiliar with the term PL still spoke in ways that reflected its principles, describing the importance of long-term health habits and enjoyment. These intentions demonstrate that holistic perspectives are present, even if not under the explicit label of PL. Many of the participants stated that they teach the reasons behind their choice of activities, such as explaining why warming up matters or highlighting the importance of recovery. Those examples illustrate that knowledge was understood as an enabler for autonomy although there was a limited in-practice application according to their practical accounts. Indeed, for most teachers, knowledge transmission was framed in traditional, teacher-centred ways in which pupils received explanations but rarely opportunities to apply them independently. The autonomy was only connected to small tasks, such as leading warm-ups or choosing a game at the end of class. Nonetheless, teachers with international experience managed to provide more meaningful accounts of autonomy-based practice. For instance, T11 stated “…I also try to incorporate elements of choice and autonomy, allowing students to make some decisions about the activities or challenges they want to pursue”, while T20 added “…I strive to stimulate them to show some independency and generate ideas, thereby practising creativity which they apply later.”

The development of confidence and competence also emerged as one of the communicated intentions across the interviews. Teachers consistently described wanting to motivate students, encourage participation, and help them build a positive relationship with physical activity. Some of them explained strategies to support weaker pupils, such as focusing more of their time on them to ensure the pupil's successful adoption of skills or celebrating every step of the pupil's progress to build up their confidence. Teachers also linked competence with broader life goals, speaking about short-term mastery of technical details, medium-term application of knowledge in daily life, and long-term adoption of physical activity into lifelong habits. However, competence was often framed narrowly around technical performance and discipline. Lesson descriptions revealed that most teachers relied on structured drills and repetitive practice to establish correct techniques, with little space or time provided for pupils' self-exploration. Such approaches demonstrate a partial adoption of PL principles—confidence and competence were definitely valued, but the methods in achieving these outcomes remained very traditional. Again, internationally experienced teachers described more flexible and student-centred approaches, highlighting reflection and inquiry as ways to develop both competence and independence. T5's account illustrates this well: “…I want them to understand movement as something fun and useful for their lives, so I often design activities where they can apply ideas and solve problems, not just copy technique”. By contrast, the predominant narrative is better captured by the T18's account stating that “Lessons typically follow a model including introduction, main part, and conclusion, and are focused on execution technique, tactics, and structured content, unless we have thoroughly mastered a significant portion of the material, which then allows for free activities”.

Overall, the findings reveal a strong intention–practice gap. Serbian PE teachers expressed philosophies that resonated with PL, often highlighting health, lifelong engagement, and pupil motivation. Yet, their practical descriptions defaulted to the traditional phased structure, with teacher-led, performance-focused instruction. Only a minority, primarily those exposed to international curricula, showed more consistent alignment between PL-related philosophies and practice. These teachers incorporated student choice, creativity, and inquiry-based learning into their teaching thereby embodying PL both in theory and practice. For the majority of teachers, however, while intentions were often positive and holistic, their delivery remained constrained by traditional methods, suggesting that systemic support, curriculum reform, and professional development are needed to bridge the gap.

### 3.2 Pre-service teachers

Interviews with pre-service teachers revealed data and themes broadly similar to those produced by in-service teachers. Here again, PL-related intentions contrasted with traditional, technique-centred lesson descriptions. Table 5 presents pre-service teachers' themed responses, clearly highlighting their intended goals and lesson delivery.

**Table 5:** Themes, sub-themes, and illustrative quotes in relation to physical literacy elements among Serbian pre-service physical education teachers.

Theme	Sub-theme	Illustrative quotes (philosophy)	Illustrative quotes (practice)
Holistic philosophy and physical literacy	Holistic intentions	“My philosophy in working with children is to first focus on their overall upbringing and personality development, and only later on their sporting abilities.” (T25) “PE should nurture not only physical abilities but also mental and social skills, creating a positive atmosphere for growth” (T24)	“A typical session begins with warm-up, main technical part, and ends with stretching or light activity.” (T21) “If pupils complete the main part well, they can get 10 minutes of free play at the end.” (T23) “Session is organised with clear phases: warm-up, drills, stretching, and discussion” (T25) “The goal is that after class or training they feel refreshed, in a good mood, a little tired but full of energy” (T21) “Every class should begin with 5–10 minutes of introduction, then the prepared main part, and end with evaluation and relaxation” (T24)
	Healthy habits and wellbeing	“The goal is for children to develop healthy habits through sport and understand the importance of physical activity for life” (T27) “My philosophy is about instilling lifelong healthy practices in students and clients” (T30)	
Enhancing pupils’ knowledge and autonomy	Lifelong application	“Short-term I want them to enjoy and feel good after class, medium-term to develop a love of activity, and long-term to adopt lifelong exercise habits.” (T21) “The focus should be on transferring healthy lifestyle habits related to physical activity” (T27)	“Cool down is stretching or some light activity with conversation about what was done in the class.” (T21) “Discipline with no delays, proper equipment, listening carefully, and following set tasks” (T27) “At the end of class there should be a concluding part with evaluation of what was done and possible application of that knowledge” (T24)
	Knowledge beyond sport	“It is important to develop emotional and social skills through play, not just physical skills” (T24) “Sessions should improve confidence, teamwork, and communication as much as physical abilities” (T23)	
Improving pupils’ confidence and competence	Motivation and encouragement	“I want to motivate pupils positively, supporting them through rewards and encouragement, while using discipline when necessary.” (T25) “Pupils should feel satisfied and proud of their progress afterwards” (T22)	“Motivation comes from progress itself, while discipline is ensured through rewards and punishments.” (T21) “It is important that children leave smiling and that I receive feedback from parents showing they are satisfied” (T24) “Sessions are focused on proper technical execution within the main part.” (T29) “The main part is devoted to technical drills and improvement of motor abilities” (T23)
	Competence and skill development	“Correct technique must be taught to ensure progress.” (T27) “Children should learn skills from many sports to broaden their motor knowledge” (T30)	

As with the in-service teachers' data, most pre-service participants had not encountered the concept of PL formally during their studies. A small number reported having heard of the term, though their descriptions were very limited. When asked to describe what PL might mean, most defined it in terms of motor competence reflecting the development of physical abilities. Despite this emphasis, their stated philosophies largely reflected the core dimensions of PL. Many highlighted the importance of shaping children into “good people” before skillful athletes, instilling healthy habits, and creating positive, supportive environments. T25's philosophy encapsulated this clearly, “My philosophy in working with children is to first focus on their overall upbringing and personality development, and only later on their sports abilities”. Others framed their philosophy around the development of lifelong routines, describing short-term aims of enjoyment, medium-term goals of fostering love for physical activity, and long-term objectives of establishing physical activity as a lifelong behaviour. These intentions resonate strongly with PL's holistic and life-course perspectives. However, the sample's descriptions of their practices once again revealed a reliance on the traditional pre-planned phased lesson format. The structure of warm-ups, drill-based main phases, and final stretching or short games dominated their responses. As T21 described, “…typical session begins with warm-up, (followed by) the main technical part, and ends with stretching or light activity.” In many cases, games or play time were explicitly framed as a reward for completing technical tasks rather than a learning tool in of itself.

When considering knowledge and autonomy, pre-service teachers again articulated intentions that reflected PL dimensions but did not align with their practice descriptions. They spoke about encouraging pupils to reflect on their progress, learn lessons that would apply beyond school, and develop emotional, social, and cognitive skills alongside motor competence. Some described the importance of pupils feeling positive and less stressed after sessions, suggesting wellbeing as a learning outcome. Many also mentioned evaluation and discussion at the end of lessons as a way of reinforcing knowledge. Practically, however, knowledge transmission was described largely in teacher-led ways, and the facilitation of pupil autonomy remained limited to providing narrow tasks such as choosing a game or leading a warm-up. Indeed, the discrepancy between the stated intention and practical execution was striking in some instances. For example, T27 mentioned that “the focus should be on transferring healthy lifestyle habits related to physical activity” but later explained that “discipline with no delays, proper equipment, listening carefully, and following set tasks” must be emphasised.

Motivation and confidence were commonly mentioned, with many referring to the use of positive feedback, enthusiasm, rewards, and sanctions. Some distinguished between children and recreational adults, describing rewards and punishments as suitable for the former, while presenting progress itself as the key motivator for the latter. In practical terms, engagement was sustained largely through discipline, order, and technical mastery with positive experiences acknowledged, but generally positioned as by-products of correct execution. Competence was predominantly framed around technical mastery, with correct performance emphasised as essential for development and injury prevention. The testing of motor skills was also frequently highlighted as a central means of monitoring progress and guiding lesson planning. Again, the claims such as, “It is important that children leave smiling and that I receive feedback from parents showing they are satisfied” (T24) were rarely supported by the practical delivery descriptions that were often “…focused on proper technical execution within the main part” (T29), while “…devoted to technical drills and improvement of motor abilities” (T23), with the claims that “correct technique must be taught to ensure progress” (T27). These accounts illustrate the strength and persistence of performance- and technique-based approaches, even when participants described philosophies that stressed holistic development. Notably, the term “discipline” emerged consistently across all of these interviews and appeared to be strongly emphasised as a key expectation of pupils, reinforced through adherence to rules and respect for the teacher in order to ensure lessons ran smoothly. While such approaches supported order and structure, they also positioned authority and rule-following at the centre of engagement, leaving limited space for autonomy, negotiation, or student-led contributions.

In summary, the findings from pre-service teachers' data mirror that from the in-service cohort. Many expressed philosophies resonating strongly with PL, particularly around holistic development, health, motivation, and lifelong engagement. Nevertheless, their descriptions of practice were shaped by structured, teacher-led lessons emphasising drills, technique, and discipline. Play and autonomy seemed peripheral to the aim of the lesson, often used as motivational tools rather than pedagogical approaches. This data would seem to demonstrate that the intention–practice gap emerges early in one's formation of a professional identity. In the Serbian context, this is clearly happening during the initial teacher education at university level, where PL-related philosophies may be present but rarely translated into aligned professional practice.

## 4 Discussion

This study examined how Serbian in-service and pre-service PE teachers perceive and apply PL in their professional practice. Across both groups, awareness of PL as a defined construct was limited, yet participants frequently expressed holistic intentions consistent with its principles, emphasising health, motivation and the development of lifelong physical activity habits. Most practical accounts, however, revealed a strong reliance on traditional, teacher-led formats centred on discipline and technical performance, with autonomy and creativity largely absent or confined to minor tasks such as leading warm-ups or choosing an end-of-class game. While a small subset of teachers with international experience described more student-centred approaches, most participants, particularly the pre-service cohort, continued to equate competence with technical execution and framed motivation in terms of discipline or external rewards. Together, these findings highlight a clear PL intention–practice gap and point to systemic challenges in embedding PL within Serbian PE.

Participants in our study largely failed to recognise PL as a clearly defined concept, with many admitting they had never encountered the term. Nevertheless, most were able to describe aspects of PL in their own words, even though very few could articulate it in its entirety. Their explanations tended to emphasise physical competence and motor skills, followed by an understanding of the benefits of physical activity and the importance of lifelong engagement. These findings align with research from Canada and Australia, where PE teachers, similarly, demonstrated only a partial understanding of PL, most often associating it with physical competence and sustained participation in activity (23, 24). Such limitations may be attributed both to variations in how PL is conceptualised across different contexts (4–8) and to the relatively recent emergence of the concept, which limits its full comprehension amongst PE teachers globally (25). In Eastern Europe, the absence of PL in teacher education and continuing professional development (CPD) appears especially pronounced. A cross-European study showed that Central and Eastern European countries scored the lowest in alignment with PL and differed significantly from their Northern European counterparts (13). While Northern European curricula explicitly connect PL to self-organisation and balanced physical and psychological health, Central and Eastern European curricula are more strongly rooted in sports performance and achievement (13). Within this regional context, most PL-related research has been conducted in Croatia (26, 27), whereas Serbia has seen very limited academic engagement with the concept. It is therefore unsurprising that the majority of PE teachers in our study reported little to no formal PL exposure. Pre-service teachers' findings mirrored that from in-service peers. Despite most not encountering the term before, they were able to conceptualise it with their own words, largely reflecting the core principles, though usually emphasising physical competence just like the in-service cohort. They clearly lacked the knowledge of how to align their practice to PL. This supports research suggesting that, even when PL can be articulated, pre-service teachers often lack the pedagogical skills to effectively foster its development in children (28, 29).

In general, teachers in this study expressed a broadly holistic philosophy that extended beyond the core elements of PL, highlighting the sociological, physical, and psychological benefits of PE as a subject. This resonates with findings from Anglo-Saxon contexts, where QPE is conceptualised as integrating PL-informed pedagogies with creativity, critical thinking, and empathy (30). Yet, PL itself remains a loosely understood construct even internationally, frequently interpreted in holistic but competence-focused terms (23–25). Our participants often stated that they sought to support the cognitive domain by enhancing pupils' knowledge of physical activity, including its value, rationale, and application in daily life. However, it was less clear how such intentions were embedded within their curricula, as few mentioned these aspects when describing their practical lessons. This tendency to frame health-related aims in theoretical rather than practical terms is not unexpected. For example, in the study by Korp et al. (31) with Swedish PE teachers, health was presented as a central concept of PE, but classroom practices offered limited evidence of how it was enacted, particularly as children may be less receptive to abstract health messages at younger ages. Indeed, even though PE can significantly support cognitive development in students (32), the teaching methods underpinning these effects remain insufficiently defined (32). Pre-service teachers in our investigation showed similar tendencies—while they spoke of promoting healthy habits and long-term routines, while using the end of lessons for reflection, their practical accounts were almost exclusively centred on structured phases, drills, and discipline, leaving little space for student autonomy or inquiry. This suggests that the gap between holistic intentions and aligned practice may begin during initial teacher education as teachers become socialized into a profession that is yet to fully embrace PL and more holistic pupil-centered approaches to teaching.

Regarding the development of pupils' confidence and competence to be active, this area was not strongly supported by our cohort's data about their practice. There is no question that contemporary pedagogical models such as the Sport Education Model, game-based approaches, and adventure-oriented activities are widely recognised as hallmarks of high-quality PE (33–35), offering clear advantages over traditional methods, which could even undermine self-esteem (34), a factor critical for sustaining motivation (36). Despite acknowledging the importance of motivation, most participants relied on traditional instructional formats characterised by rigidly structured lessons and games reserved for the end of class, often framed as a form of reward. This discrepancy between stated philosophies and reported practice emerged as one of the central findings from this paper. While many claimed to encourage students to adopt lifelong healthy habits and foster a motivation to be active for life, their lesson descriptions reflected approaches largely inconsistent with PL principles. Notably, only the small subset of teachers with international teaching experience (*n* = 4) provided accounts of lessons that incorporated greater student autonomy, creativity, and collaboration, aligning their theoretical understanding of PL with their classroom practice. Younger teachers also occasionally demonstrated leanings towards PL-informed approaches, whereas older teachers tended to describe class structures that adhered more closely to traditional, performance-oriented models. This is a well recognised outcome as older teachers often report being less prone to reconsider their approaches that are strongly embedded in their contextual beliefs (37, 38). Nonetheless, several older teachers described shifts away from teacher-centred methods toward more pupil-centred pedagogy as a result of accumulated experience. This aligns with research showing that experienced teachers can sometimes adopt more child-centred strategies compared to novices, but such research also highlights the role of professional socialisation and local conditions in shaping pedagogical enactment (39).

Pre-service teachers demonstrated the contradictory tendency of supporting ideas around promoting children's lifelong physical activity whilst consistently placing discipline and control at the centre of their lesson expectations. Comparative research suggests that, despite believing in the effectiveness of PE to facilitate changes in pupil behaviour more than their in-service peers (40, 41), pre-service teachers often require explicit preparation to translate such intentions into autonomy-supportive practice (42). In line with this, pupil autonomy in our study was largely absent from practical descriptions, appearing only in limited forms such as leading warm-ups, choosing a final game, or receiving time for reflection at the end of class. Notably, although autonomy-oriented curricula are associated with higher levels of skill attainment and perceived competence (43), especially for pupils with limited motivation (44), not all students feel comfortable when required to make decisions (44, 45). This is an important consideration for teacher training to reflect upon in order to enable future teachers to provide meaningful opportunities for student independence and confidence formation rather than designing activities that may undermine it (42, 44, 45). CPD on PL and its effective delivery in practice is essential to ensure sustained impact on students' affective learning outcomes (24, 46–48). Stakeholders should, therefore, prioritise enhancing teachers' own PL and self-efficacy in order to deliver QPE and, in turn, hopefully strengthen pupils' own PL development (49, 50). Such work is crucial in Serbia if PL is to be meaningfully integrated in ways that are consistent with the country's wider physical culture (51).

This study is not without limitations. Although participants were drawn from different urban areas in Serbia, the sample did not include rural regions, which may restrict the findings' transferability to the wider national context. In addition, the reliance on self-reported practices means classroom realities may not be fully captured, and the absence of observational data limits the ability to verify alignment between teachers' stated philosophies and their practical delivery. Nevertheless, the inclusion of 31 participants covering both in-service and pre-service PE teachers across elementary and secondary levels, with variation in sex, experience, school level and school type, represents a highly heterogeneous cohort for qualitative research. This provided an insightful and nuanced picture of how PL is understood and enacted in the Serbian context, contributing new knowledge in a region where the concept has received little academic attention to date.

## 5 Conclusion and future directions

This study is the first to explore how both in-service and pre-service Serbian PE teachers understand and apply PL in their philosophy and practice. Findings revealed limited awareness of PL as a clearly defined construct, though many participants expressed holistic intentions consistent with its principles. Across both groups, a clear gap emerged between the teachers' expressed teaching philosophy and their applied practice—while teachers frequently emphasised health, motivation, and lifelong engagement, lesson descriptions were largely rooted in traditional, teacher-led formats centred on drills, discipline, and technical performance. Only a minority of teachers, primarily those with international experience, described more student-centred practices that seemed well aligned with PL principles. These findings clearly underline the need for curriculum reform and targeted professional development in Serbia if PL is to be meaningfully embedded into Serbian PE, enabling pupils to experience richer opportunities for autonomy, confidence, and the development of more positive dispositions toward lifelong participation in recreational sport and physical activity. Future research should continue to investigate systemic enablers and barriers, including how pre-service preparation can be strengthened to bridge the intention–practice gap from the outset of teachers' careers. It should also widen its stakeholder focus and scope to explore the views and experiences of the PE teacher trainers, parents and the children themselves. Studies could combine our interpretive qualitative approach with quantitative data on teacher and pupil behaviours as well as attitude, motivation and lifestyle survey scales. This would provide a fuller picture of the Serbia's contextual-cultural picture to comprehensively inform a theory of change for the PE teaching profession.

## Data Availability

The raw data supporting the conclusions of this article will be made available by the authors, without undue reservation.

## References

[1] Whitehead M. Physical Literacy: Throughout the Lifecourse. London: Routledge (2010). 256.

[2] Carl J Bryant A Edwards L Bartle G Birch J Christodoulides E Physical literacy in Europe: the current state of implementation in research, practice, and policy. J Exerc Sci Fit. (2022) 21(1):165–76. 10.1016/j.jesf.2022.12.003 36688001 PMC9827378

[3] Jurbala P. What is physical literacy, really? Quest. (2015) 67:367–83. 10.1080/00336297.2015.1084341

[4] Bailey R Glibo I Koenen K Samsudin N. What is physical literacy? An international review and analysis of definitions. Kinesiol Rev. (2023) 12(3):247–60.

[5] IPLA. International Physical Literacy Association—About. Loughborough: IPLA (2022). Available online at: https://www.physical-literacy.org.uk/about/?v=3a52f3c22ed6 (Accessed August 13, 2025).

[6] Li MH Whitehead M Green N Ren H Cheng CF Lin LLC Operationally defining physical literacy in Chinese culture: results of a meta-narrative synthesis and the panel’s recommendations. J Exerc Sci Fit. (2022) 20(3):236–48. 10.1016/j.jesf.2022.04.003 35646130 PMC9117885

[7] Sport England. Physical Literacy Consensus Statement for England published. Sport England.. September 28, 2023. Available online at: https://www.sportengland.org/news-and-inspiration/physical-literacy-consensus-statement-england-published (Accessed August 13, 2025).

[8] Sport Australia. The Australian Physical Literacy Framework. Belconnen: Sport Australia (2019). Available online at: https://www.sportaus.gov.au/__data/assets/pdf_file/0019/710173/35455_Physical-Literacy-Framework_access.pdf (Accessed August 13, 2025).

[9] Young L Alfrey L O’Connor J. Moving from physical literacy to co-existing physical literacies: what is the problem? Eur Phy Educ Rev. (2023) 29(1):55–73. 10.1177/1356336X221112867

[10] Chair(s), Carl J Elsborg P. S2-1 physical literacy in Europe. Eur J Public Health. (2023) 33(Suppl 1):ckad133.009. 10.1093/eurpub/ckad133.009

[11] Carl J Mazzoli E Mouton A Sum RKW Singh A Niederberger M Development of a global physical literacy (GloPL) action framework: study protocol for a consensus process. PLoS One. (2024) 19(8):e0307000. 10.1371/journal.pone.0307000 39133681 PMC11318864

[12] Carl J Barratt J Arbour-Nicitopoulos KP Barnett LM Dudley DA Holler P Development, explanation, and presentation of the physical literacy interventions reporting template (PLIRT). Int J Behav Nutr Phys Act. (2023) 20:21. 10.1186/s12966-023-01423-3 36805731 PMC9938627

[13] Carl J Goss H Lundvall S Pavlova I Algurén B Antala B Compatibility of physical education curricula with physical literacy across 40 European countries. J Curric Stud. (2025) 1–21. 10.1080/00220272.2025.2523436

[14] Sports in Serbia: A Closer Look–TheFlags.org. (2024). Available online at: https://theflags.org/sports-in-serbia-a-closer-look/ (Accessed May 20, 2025).

[15] Cvetković B Cvetković M Petrušič T Đorđić V Bubanj S Popović B Nutrition and physical activity behavior in 11–14-year-old schoolchildren in Serbia. Children (Basel). (2021) 8(8):625. 10.3390/children8080625 34438516 PMC8394318

[16] Jovanović M Minić V. Teachers of physical education on improving the quality of teaching with continuous adjustments to the curricula. FU Phys Ed Sport. (2019):651. 10.22190/FUPES180208059J

[17] Radisavljević-Janić S Milanović I. Physical education in the republic of Serbia. Fizička Kultura. (2019) 73(1):61–71. 10.5937/fizkul1901061R

[18] Csordás-Makszin Á Spray CM Berki T Hamar P Karsai I Soós I. Antecedents of physical education teachers’ motivational strategies in central Europe. Eur Phy Educ Rev. (2025) :1356336X251337075. 10.1177/1356336X251337075

[19] Rio CJ Saligan LN. Understanding physical activity from a cultural-contextual lens. Front Public Health. (2023) 11:1223919. 10.3389/fpubh.2023.1223919 37601221 PMC10436229

[20] Grecic D. The epistemological chain: a tool to guide TNE development. J Stud Int Educ. (2022):10283153221145078. 10.1177/10283153221145078

[21] Braun V Clarke V. One size fits all? What counts as quality practice in (reflexive) thematic analysis? Qual Res Psychol. (2021) 18(3):328–52. 10.1080/14780887.2020.1769238

[22] Tracy SJ. Qualitative quality: eight “big-tent”. Criteria for excellent qualitative research. Qual Inq. (2010) 16(10):837–51. 10.1177/1077800410383121

[23] Essiet IA Warner E Lander NJ Salmon J Duncan MJ Eyre ELJ Exploring Australian teachers’ perceptions of physical literacy: a mixed-methods study. Phys Educ Sport Pedagogy. (2024) 29(1):18–37. 10.1080/17408989.2022.2028760

[24] Stoddart AL Humbert ML. Teachers’ perceptions of physical literacy. Curric J. (2021) 32(4):741–57. 10.1002/curj.107

[25] Yin H Dev RDO Soh KG Li F Lian M. Assessment and development of physical education teachers’ physical literacy: a systematic review. PLoS One. (2024) 19(7):e0307505. 10.1371/journal.pone.0307505 39024325 PMC11257255

[26] Rajkovic Vuletic P Gilic B Zenic N Pavlinovic V Kesic MG Idrizovic K Analyzing the associations between facets of physical literacy, physical fitness, and physical activity levels: gender- and age-specific cross-sectional study in preadolescent children. Educ Sci. (2024) 14(4):391. 10.3390/educsci14040391

[27] Gilic B Sekulic D Munoz MM Jaunig J Carl J. Translation, cultural adaptation, and psychometric properties of the perceived physical literacy questionnaire (PPLQ) for adults in Southeastern Europe. J Public Health (Berl). (2025). 10.1007/s10389-025-02428-x40917130

[28] Stoddart AL Selanders KP. Preparing for physical literacy: exploring pre-service teachers’ training and understanding. Teach Teach Educ. (2022) 120:103886. 10.1016/j.tate.2022.103886

[29] Curtin University, Dinham J Williams P. Developing children’s physical literacy: how well prepared are prospective teachers? AJTE. 2019 44(6):53–68. 10.14221/ajte.2018v44n6.4

[30] Houser N Kriellaars D. “Where was this when I was in physical education?” physical literacy enriched pedagogy in a quality physical education context. Front Sports Act Living. (2023) 5. 10.3389/fspor.2023.1185680/full 37305659 PMC10249748

[31] Korp P Quennerstedt M Barker D Johansson A. Making sense of health in PE: conceptions of health among Swedish physical education teachers. Health Educ. (2023) 123(2):79–92. 10.1108/HE-11-2022-0086

[32] Cho O Choi W Shin Y. The effectiveness of school physical education on students’ cognitive competence: a systematic review and meta-analysis. J Sports Med Phys Fit. (2022) 62(4):575–84. 10.23736/S0022-4707.21.11796-7 35350096

[33] Dudley D Mackenzie E Van Bergen P Cairney J Barnett L. What drives quality physical education? A systematic review and meta-analysis of learning and development effects from physical education-based interventions. Front Psychol. (2022) 13. 10.3389/fpsyg.2022.799330 35846697 PMC9280720

[34] Bessa C Hastie P Rosado A Mesquita I. Sport education and traditional teaching: influence on students’ empowerment and self-confidence in high school physical education classes. Sustainability. (2021) 13(2):578. 10.3390/su13020578

[35] Koszałka-Silska A Korcz A Wiza A. The impact of physical education based on the adventure education programme on self-esteem and social competences of adolescent boys. Int J Environ Res Public Health. (2021) 18(6):3021. 10.3390/ijerph18063021 33804140 PMC8000378

[36] Estevan I Bardid F Utesch T Menescardi C Barnett LM Castillo I. Examining early adolescents’ motivation for physical education: associations with actual and perceived motor competence. Phys Educ Sport Pedagogy. (2021) 26(4):359–74. 10.1080/17408989.2020.1806995

[37] Dockerty F Pritchard R. Reconsidering models-based practice in primary physical education. Education. (2023):1–12. 10.1080/03004279.2023.2263470

[38] Fernández Rivas M Espada M. Importance of teaching styles in physical education classes: perceptions according to age, teachers’ degree and school ownership. J Phys Educ Sport. (2021) 21:3294–302. 10.7752/jpes.2021.s6437

[39] Graham G Manross M Hopple C Sitzman T. Novice and experienced children’s physical education teachers: insights into their situational decision making. J Teach Phys Educ. (1993) 12(2):197–214. 10.1123/jtpe.12.2.197

[40] Faculty of Sport Sciences, Balıkesir University, Balcı T Yanık M. In-service and pre-service physical education teachers’ levels of belief in education: a comparative study. EPASR. (2021) 16(4):30–48. 10.29329/epasr.2021.383.2

[41] Iannucci C MacPhail A. The effects of individual dispositions and workplace factors on the lives and careers of physical education teachers: twelve years on from graduation. Sport Educ Soc. (2017) 24. 10.1080/13573322.2017.130717532848505

[42] Großmann N Fries S Wilde M. Is the practice of autonomy support the missing element in teacher training at university? A study on the effects of an intervention based on self-determination theory on biology preservice teachers’ knowledge, beliefs, and intentions. Front Psychol. (2023) 14:1279771. 10.3389/fpsyg.2023.1279771 38106398 PMC10722232

[43] Hastie PA Rudisill ME Wadsworth DD. Providing students with voice and choice: lessons from intervention research on autonomy-supportive climates in physical education. Sport Educ Soc. (2013). 10.1080/13573322.2012.70120325960685

[44] Holt AD Smedegaard S Pawlowski CS Skovgaard T Christiansen LB. Pupils’ experiences of autonomy, competence and relatedness in “move for well-being in schools”: a physical activity intervention. Eur Phy Educ Rev. (2019) 25(3):640–58. 10.1177/1356336X18758353

[45] Zach S Yanovich E. Autonomy, choice, and pupils’ motivation—are they really related? Adv Phys Educ. (2015) 5(2):84–93. 10.4236/ape.2015.52011

[46] Effects of teachers’ participation in continuing professional development on students’ perceived physical literacy, motivation and enjoyment of physical activity. Revista de Psicodidáctica (English ed). (2022) 27(2):176–85. 10.1016/j.psicoe.2022.05.003

[47] Telford RM Olive LS Keegan RJ Keegan S Telford RD. Teacher and school outcomes of the physical education and physical literacy (PEPL) approach: a pragmatic cluster randomised controlled trial of a multicomponent intervention to improve physical literacy in primary schools. Phys Educ Sport Pedagogy. (2021) 26(1):79–96. 10.1080/17408989.2020.1799965

[48] Hernaiz-Sánchez A Villaverde-Caramés EJ González-Valeiro M Fernández-Villarino MA. Physical literacy and teacher training: pilot study. Educ Sci. (2021) 11(2):42. 10.3390/educsci11020042

[49] Sum RKW Morgan K Ma MMS Choi SM. The influence of a customized continuing professional development programme on physical education teachers’ perceived physical literacy and efficacy beliefs. Prospects. (2021) 50(1):87–106. 10.1007/s11125-020-09471-4

[50] Choi SM Sum R Wallhead T Ha A Sit C Shy DY Preservice physical education teachers’ perceived physical literacy and teaching efficacy. J Teach Phys Educ. (2021) 40:146–56. 10.1123/JTPE.2019-0076

[51] Irmansyah J Susanto E Lumintuarso R Sugiyanto F Syarif A Syah H. Physical literacy in the culture of physical education in elementary schools: Indonesian perspectives. Int J Hum Mov Sports Sci. (2021) 9:929–39. 10.13189/saj.2021.090514

